# Vitamin D and IL-10 Deficiency in Preterm Neonates With Bronchopulmonary Dysplasia

**DOI:** 10.3389/fped.2018.00246

**Published:** 2018-09-07

**Authors:** Xiaonan Mao, Jie Qiu, Li Zhao, Junjie Xu, Jiao Yin, Yang Yang, Mingshun Zhang, Rui Cheng

**Affiliations:** ^1^Department of neonates, Children's Hospital of Nanjing Medical University, Nanjing, China; ^2^Department of Immunology, Nanjing Medical University, Nanjing, China

**Keywords:** bronchopulmonary dysplasia, preterm, vitamin D, IL-10, blood, inflammation

## Abstract

**Introduction:** Vitamin D deficiency and inflammation are involved with bronchopulmonary dysplasia (BPD) in preterm neonates; however, the clinical evidence still remains scarce. We hypothesized that vitamin D and inflammatory cytokines may be risk factors for BPD in infants.

**Methods:** Preterm infants born between 28 and 31 weeks' gestation were recruited between January 2016 and 2017. Blood samples were all collected at corresponding time points. Vitamin D was measured using an automatic biochemical analyzer, and inflammatory cytokines (TNF-α, IL-1β, IL-6, and IL-10) were measured using ELISA.

**Results:** The baseline characteristics for preterm infants without BPD (non-BPD control, *n* = 20) or with BPD (*n* = 19) were similar. In the blood samples collected 24-h post birth, vitamin D was significantly reduced in the BPD neonates (non-BPD vs. BPD, 28.96 ± 3.404 vs. 17.99 ± 2.233 nmol/l, *p* = 0.0134). Inflammatory cytokines TNF-α, IL-1β, and IL-6 were comparable in both groups. The anti-inflammatory cytokine IL-10, however, was significantly decreased in 24-h blood samples from BPD preterm infants (non-BPD vs. BPD, 44.61 ± 10.48 vs. 11.64 ± 2.351 pg/ml, *p* = 0.0054). In the BPD infants with mild or moderate disease, vitamin D deficiency was quite similar. IL-10 deficiency, however, was more aggravated in the BPD infants with moderate disease. No changes in Vitamin D or cytokines (TNF-α, IL-1β, IL-6, and IL-10) were observed for blood samples collected 2 or 4 weeks after birth.

**Conclusion:** In our pilot study, Vitamin D and IL-10 levels at 24-h of life were risk factors for the development of BPD in very preterm infants.

## Introduction

Bronchopulmonary dysplasia (BPD) is a major form of chronic lung diseases in preterm infants and is primarily due to respiratory distress syndrome (RDS) (1). Arguably, mechanical ventilation and long-term use of oxygen may contribute to BPD onset. However, with multiple strategies to provide ventilation and oxygen therapy, the incidence of BPD has not decreased. Although great advances have been made in animal BPD models, clinical research to explore the pathogenesis and treatment of BPD is imperative.

Vitamin D deficiency is common in preterm infants (2). However, the evidence of vitamin D deficiency in human fetal and neonatal lung diseases is insufficient (3). Çetinkaya et al. found that lower maternal and neonatal vitamin 25-OHD levels were associated with BPD development in preterm infants (gestational ages <32 weeks) (4). Koroglu et al. reported that the vitamin D receptor polymorphism was associated with an increased frequency of BPD (5). In contrast, KE Joung et al. recorded that low vitamin D was common among preterm infants at birth; however, they did not detect any association between vitamin D status and pulmonary or other morbidities of prematurity (6). Prem Fort et al. also reported that vitamin D deficiency was frequent in extremely preterm infants (gestational ages 23–27 weeks); yet, vitamin D supplementation did not improve clinical outcomes (7). In sum, the role of vitamin D for BPD development in preterm infants is still controversial.

Antenatal exposure to inflammatory cytokines is an independent risk factor for BPD pathogenesis. In the amniotic fluid from BPD infants, IL-1β, IL-6, and IL-8 were significantly increased (8). IL-6, IL-8, and IL-10 cord blood levels were also significantly altered in preterm BPD newborns (9). In the tracheal aspirate samples, IL-6, IL-8, IL-10, and TNF-α were significantly altered in BPD infants (10). Previously, we demonstrated that vitamin D in preterm BPD infants (gestational ages <34 weeks) was significantly decreased (11). In the present study, we expanded our research and explored whether vitamin D and inflammatory cytokines (TNF-α, IL-1β, IL-6, and IL-10) in venous blood could be risk factors for BPD in preterm infants.

## Patients and methods

### Preterm infants

Preterm infants with RDS born at gestational age ≤ 32 weeks were admitted to the Neonatal Intensive Care Unit at Children's Hospital of Nanjing Medical University and were enlisted in the study between January 2016 and 2017. The infants were diagnosed with BPD according to the workshop definition by the National Institutes of Child Health, the Human Development/National Heart, Lung, and Blood Institute and the Office of Rare Diseases (12). Preterm infants without BPD were randomly selected for the control group. To reduce the difference in the baseline characteristics, only preterm RDS infants between 28 and 31 weeks were included in the Vitamin D and cytokine quantification. Infants with severe congenital malformations, severe infection, shock, and inherited metabolic diseases were excluded from the study. Infants born to mothers with vitamin D deficiency or a severe infection were also excluded.

Preterm infants may receive vitamin D through parenteral nutrition and milk at the beginning (13). Vitamin D was added into venous nutrition at 36–48 IU/kg of vitamin D. Venous nutrition was given to infants until they could consume 140-150 ml/kg of milk. The infants were also treated with protein deep hydrolyzed milk, which contains 0.5 IU/ml of vitamin D. In the first 2 weeks, preterm infants could gradually drink 160 ml of milk each day and acquire 80 IU of vitamin D. Moreover, all preterm infants were treated with oral vitamin D (800 IU, Qd) starting at 15 days of life.

### Collection methods

(1) Routine clinical data were collected for all enrolled infants. (2) A total of 1 ml of fasting peripheral venous blood samples were taken and saved in a pro-coagulant tube each time. The 25(OH) D (represented as Vitamin D) level was measured using an automatic biochemical analyzer (type 1024, Tokyo, Japan) in nmol/l. Inflammatory cytokines (TNF-α, IL-1β, IL-6, and IL-10) were measured using an enzyme-linked immunosorbent assay (using Quantikine® ELISA kit, Minneapolis, United States) in pg/ml.

### Statistical methods

Statistical analysis was performed using Graphpad Prism 7. Quantitative data are shown as the mean ± standard error of the mean. The data between two groups were compared using the *t*-test, and the data among more than two groups were compared using ANOVA with a *post hoc* Bonferroni test. For the qualitative data, the Pearson chi-square test was performed. *P* < 0.05 was considered to be statistically significant.

## Results

### Baseline data comparison between the BPD group and the control group

In total, 67 preterm infants were recruited for this the study. Eleven infants were discharged against the advice of the hospital and were not included in further research. To reduce the baseline differences among BPD and non-BPD infants, we only performed tests on neonates between 28 and 31 weeks. The final study volume included 19 BPD infants and 20 non-BPD control newborns. As shown in Table 1, gestational age and body weight in BPD infants and non-BPD controls were comparable. Days with CPAP or days with oxygen, however, were significantly prolonged in the preterm BPD neonates. Patent ductus arteriosus (PDA) was more common in BPD patients.

**Table 1:** Clinical characteristic of the BPD and non-BPD infants.

	**non-BPD (*****n*** = **20)**		**BPD (*****n*** = **19)**		***P*-value**
	**Mean ± SEM**	***n*(%)**	**Mean ± SEM**	***n*(%)**	
**INFANT CHARACTERISTICS**					
Male gender		10 (50)		12 (63.2)	0.5231
Birth weight (grams)	1323 ± 51.85		1212 ± 50.46		0.1326
Gestational age (days)	208.5 ± 1.140		205.1 ± 1.992		0.1448
Apgar 1 min	7.895 ± 0.1509		8.053 ± 0.1617		0.4800
Apgar 5 min	8.842 ± 0.1150		8.632 ± 0.2321		0.4217
Intraventricular hemorrhage (IVH)		20(100)		19(100)	1.0000
Periventricular Leukomalacia (PVL)		20(100)		19(100)	1.0000
Necrotizing enterocolitis (NEC)		2(10.0)		5(26.3)	0.2351
Late-onset neonatal sepsis (LOS)		1(5.0)		3(15.8)	0.3416
Intrauterine growth restriction (IUGR)		5(25.0)		7(36.8)	0.5006
Mechanical Ventilation (days)	3.316 ± 0.7135		5.316 ± 0.8552		0.0809
CPAP (days)	2.750 ± 0.5572		13.05 ± 1.755		< 0.0001
Days with oxygen	6.526 ± 1.836		22.76 ± 2.892		< 0.0001
Surfactant treatment		18 (90.0)		16 (84.2)	0.6614
Patent ductus arteriosus (PDA)		3 (15.0)		10 (52.6)	0.0187
Hospitalization days	31.53 ± 5.417		52.00 ± 3.824		0.0039
**MATERNAL CHARACTERISTICS**					
Preeclampsia		5 (25.0)		8 (42.1)	0.3203
Chorioamnionitis		2(10.0)		4(21.1)	0.4075
Group B streptococcus status		0		0	1.0000
Antenatal antibiotics		5(25.0)		6(31.6)	0.7311
Antenatal steroids		11(55.0)		10(52.6)	1.0000
Delayed ligation of umbilical cord		20(100)		19(100)	1.0000
Premature rupture of membranes (PROM)		10 (50.0)		4 (21.1)	0.0958

### Vitamin D deficiency in BPD patients

**Figure 1:**
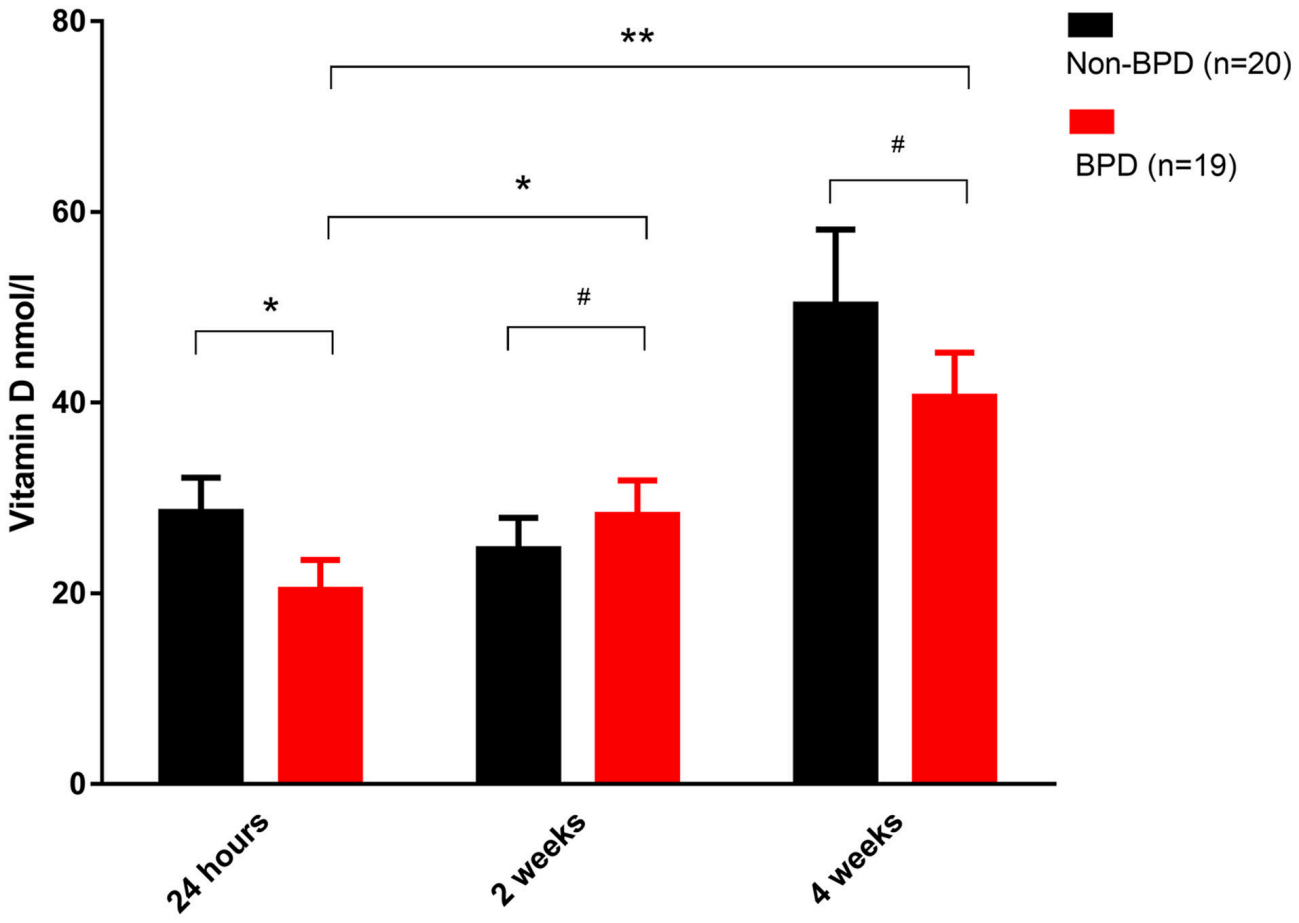
Vitamin D deficiency in the BPD patients. Venous blood samples were taken at the indicated time and plasma vitamin D was quantified. Vitamin D was significantly decreased in the 24-h samples from the BPD patients (*p* = 0.0134). In the 2 or 4 weeks samples, however, vitamin D was comparable in both groups. Compared with the 24-h samples, vitamin D in the BPD patients was significantly recovered after 2 weeks (*p* = 0.0423) or 4 weeks of therapy (*p* = 0.0007). Twenty non-BPD patients and 19 BPD patients were included in the final study. #*p* > 0.05; *p < 0.05; **p < 0.01.

Venous blood from BPD infants and non-BPD controls were collected 24-h, 2 and 4 weeks after birth. In accordance with our previous observation (14), we again recorded that the vitamin D concentration 24-h post birth was significantly reduced in BPD infants (28.96 ± 3.404 vs. 17.99 ± 2.233 nmol/l, *p* = 0.0134). After vitamin D supplementation therapy for 2 or 4 weeks, vitamin D was gradually increased either in the non-BPD controls or in the BPD patients, and the vitamin D concentration was comparable between the two groups. In BPD preterm neonates, vitamin D in the blood from 2 or 4 weeks samples was significantly higher than that in the 24-h blood samples. In sum, vitamin D deficiency was present at birth among the BPD patients and gradually recovered after diet and medical supplementation therapy (Figure 1).

### Inflammatory cytokine profile of the BPD patients

**Figure 2:**
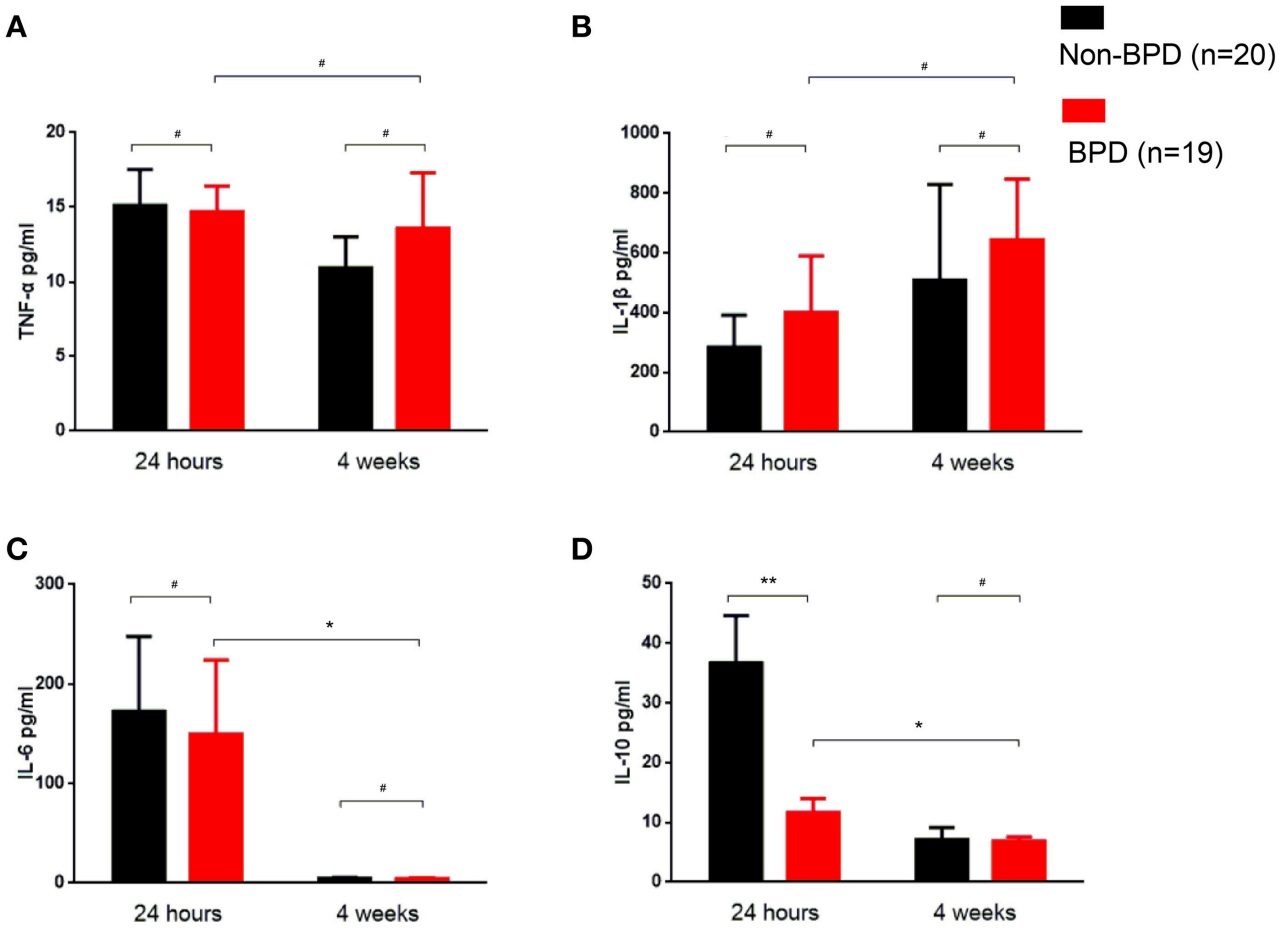
Inflammatory cytokine profiles of the BPD patients. Venous blood samples were taken at the indicated time, and cytokines were quantified using ELISA. **(A)** TNF-α was similar in 24-h and 4 weeks samples between BPD and non-BPD neonates. **(B)** IL-1β was almost invariable in the samples at different time points from BPD and non-BPD controls. **(C)** IL-6 was comparable in the 24-h samples or 4 weeks samples from BPD or non-BPD controls. Compared to the 24-h samples, the IL-6 level was significantly decreased in the 4 weeks samples of BPD patients (*p* = 0.0493). **(D)** IL-10 was significantly decreased in the 24-h samples from preterm infants with BPD (*p* = 0.0054). As observed in IL-6, IL-10 was further significantly decreased after 4 weeks of therapy (*p* = 0.0346). Twenty non-BPD patients and 19 BPD patients were included in the final study. #*p* > 0.05; *p < 0.05; **p < 0.01.

In the venous blood samples within 24-h post-birth, IL-10 was significantly decreased in the BPD preterm newborns (44.61 ± 10.48 vs. 11.64 ± 2.351 pg/ml, *p* = 0.0054). After 4 weeks of therapy, IL-10 decreased and was comparable in the BPD and non-BPD neonates. TNF-α, IL-1β, and IL-6 were similar in the 24-h and 4 weeks samples from the BPD and non-BPD neonates. Interestingly, TNF-α and IL-1β in the BPD patients were constant in the 24-h and 4 weeks samples. IL-6 and IL-10 levels in the infants with BPD were significantly decreased at 4 weeks compared to the values at 24-h. (Figure 2)

### IL-10 deficiency contributed to BPD disease severity

**Figure 3:**
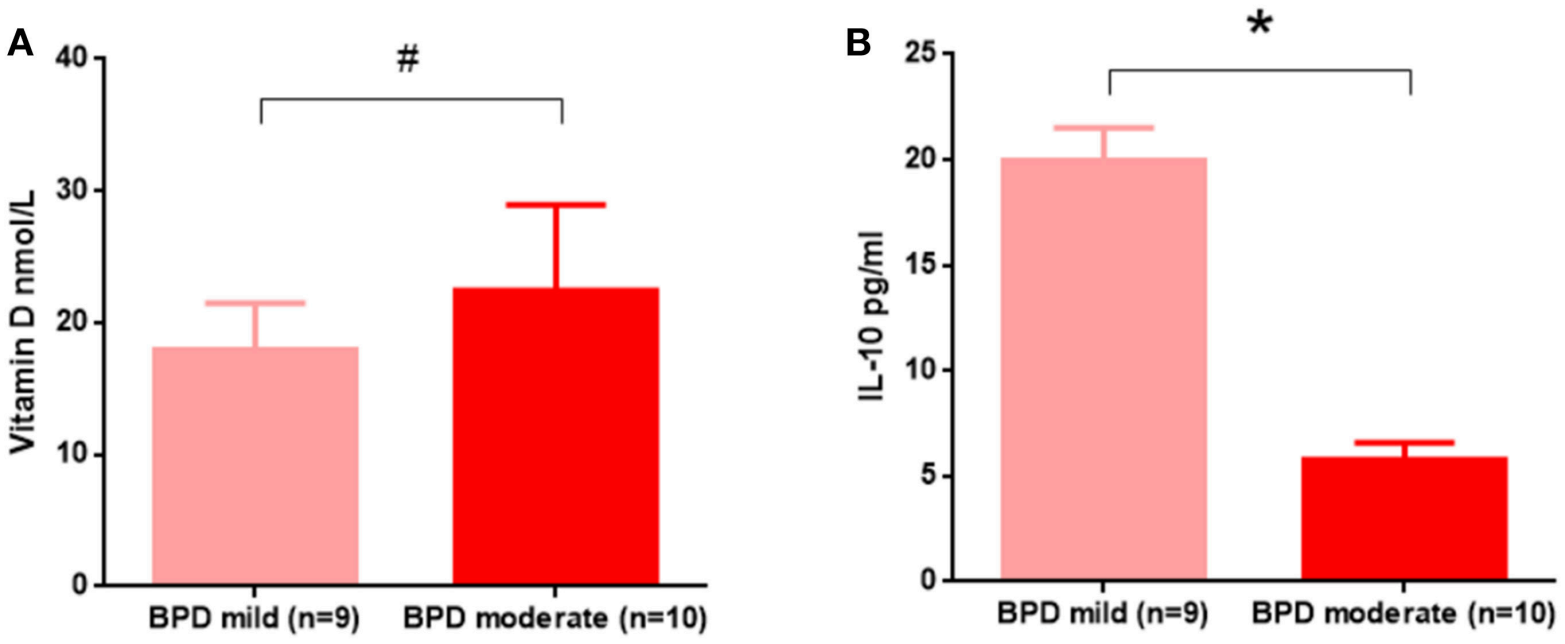
In the 24 h samples, IL-10, but not vitamin D, contributed to BPD disease severity. **(A)** Vitamin D levels were not significantly different between mild and moderate BPD infants (*p* = 0.5392); **(B)** Compared to mild BPD infants, IL-10 was significantly decreased in moderate BPD neonates (*p* = 0.0344). Nine BPD patients with mild disease and 10 BPD patients with moderate disease were analyzed in this study. #*p* > 0.05; *p < 0.05.

We observed decreased vitamin D and IL-10 levels in the 24-h blood samples from BPD preterm neonates. As shown in Figure 3, vitamin D deficiency was similar in mild and moderate BPD patients. However, IL-10 was significantly reduced in the BPD patients with moderate disease.

## Discussion

Vitamin D deficiency was observed in new-born preterm infants with BPD in our study and by other labs (4). Preterm infants may acquire vitamin D through venous nutrition and milk. Therefore, we speculated that vitamin D supplementation may help to recover the blood concentration of vitamin D in 2 weeks. However, the BPD patients still demanded more days with oxygen, suggesting that clinical outcomes had not beengreatly improved upon vitamin D therapy. Moreover, vitamin D deficiency was not associated with BPD severity, and this finding is in line with Prem Fort's observation that vitamin D supplementation did not improve BPD (7). Vitamin D may indirectly regulate the pathogenesis of BPD, i.e., via inflammatory cytokines. For example, Chen et al. treated hyperoxia-exposed animals with 1,25(OH)2D3, which significantly downregulated the expression of inflammatory cytokines and TNF-α and reduced hyperoxia-induced lung injury (15).

In the BPD infants, the inflammatory cytokines IL-1β, IL-6, TNF-α, and IL-10 levels were altered in the amniotic fluid (8), cord blood (9), and tracheal aspirate samples (10). TNF-α single nucleotide polymorphisms can predict BPD onset and severity in preterm neonates (16, 17). However, Harald Ehrhardt et al. reported that reduced rather than elevated TNF-α in tracheal aspirate samples were associated with BPD severity in BPD preterm patients (18). In the present study, the levels of the inflammatory cytokines IL-1β, IL-6, and TNF-α were similar in the venous blood within 24-h post-birth in patients with or without BPD. The anti-inflammatory cytokine IL-10, however, was significantly reduced in preterm patients with BPD. Moreover, IL-10 deficiency was associated with BPD disease severity, which was similar to previous findings in the cord blood (9). IL-10 expression was low in preterm infants (19). In the present study, the baseline characteristics between the BPD and non-BPD infants were similar. Therefore, the reduced IL-10 in patients with BPD may reflect that IL-10 was an independent risk factor for BPD.

Our study was not without limitations. First, the number of BPD patients was small. In the last year (201601–201701), a total of 4,662 term and preterm infants were admitted to the NICU, and 110 were diagnosed with BDP. Therefore, the incidence of BPD was 2.3% of all newborns. In the future, we would like to expand our research and quantify vitamin D and IL-10 in a larger number of BPD infants, especially extremely and very premature infants. In the present study, we only focused on infants born at 28 to 31 weeks. Second, due to the limited number of BPD patients, we could not further divide them into common BPD and atypical BPD patients (20). The obtained results, however, supported the hypothesis that deficiency in vitamin D and IL-10 may contribute to BPD in preterm infants. Further studies with a larger number of cases are required to assess the values of vitamin D and IL-10 in the prediction and diagnosis of BPD.

## Ethics statement

This study was performed in accordance with the recommendations of the Committee on human rights related to research involving human subjects, Faculty of Affiliated Children Hospital, Nanjing Medical University (Number: NJCH2016003). Written informed consent was obtained from the parents of the infants in this study.

## Author contributions

XM, JQ, and LZ collected and measured the biological samples. JX, JY, and YY collected the clinical data. MZ and RC designed the experiment, analyzed the data and wrote the paper.

### Conflict of interest statement

The authors declare that the research was conducted in the absence of any commercial or financial relationships that could be construed as a potential conflict of interest.
